# Ripening Indices, Olive Yield and Oil Quality in Response to Irrigation With Saline Reclaimed Water and Deficit Strategies

**DOI:** 10.3389/fpls.2019.01243

**Published:** 2019-10-09

**Authors:** Cristina Romero-Trigueros, Gaetano Alessandro Vivaldi, Emilio Nicolás Nicolás, Antonello Paduano, Francisco Pedrero Salcedo, Salvatore Camposeo

**Affiliations:** ^1^Dipartimento di Scienze Agro-Ambientali e Territoriali, Università degli Studi di Bari Aldo Moro, Bari, Italy; ^2^Department of Irrigation, Centro de Edafología y Biología Aplicada del Segura (CEBAS-CSIC), Murcia, Spain

**Keywords:** acidity, Arbosana, fruit weight, oil extractability, oleic acid, polyphenol, peroxide, total production

## Abstract

The 70% worldwide surface of olive orchards is irrigated. The evaluation of non-conventional water resources and water-saving techniques has gained importance during the last decades in arid and semiarid environments. This study evaluated the effects of irrigation with two water sources: low-cost water DEsalination and SEnsoR Technology (DESERT) desalinated water (DW) EC_w_ ∼1 dS m^−1^) and reclaimed water (RW) (EC_w _∼ 3 dS m^−1^) combined with two irrigation strategies: full irrigation (FI) (100% of ET_c_) and regulated deficit irrigation (RDI, 50% of ET_c_) on fruit yield, ripening indices, and oil yield and quality of olive trees cv Arbosana planted in Mediterranean conditions. Our results showed that RW without water restrictions increased the fruit yield by 35% due to a slight increase in the fruit weight and, mainly, to a greater fruit set than the control trees; although this did not result in a higher oil yield (g tree^−1^) since the oil content per fruit dry weight was reduced. The RDI strategy did not decrease the fruit yield despite the fact that olive weight tended to decrease, and it increased the oil yield by ∼14.5%. The combination of both stresses (RW and RDI) neither decreased the fruit yield; however, it significantly reduced oil yield (25% less in 2018) since oil content per fruit dry weight was strongly reduced (40%) compared to control trees. Both RDI treatments, regardless water source, determined acidity levels in olive paste lower than in FI treatments; however, it reduced oil extractability and fatty yield. The finding about oil quality indicated that olive exposure to RW, regardless of the water amount, decreased oil quality mainly due to the reduction of oleic acid and the increase of C18:2/C18:3 ratio and peroxides; on the contrary, both RW and RDI improved the total polyphenols. In all cases, the parameters met the legislation. In short, with appropriate management, RW and RDI have great potential to manage oil olive production; nevertheless, studies subjected to long-term use of these techniques should be experienced to ensure the sustainability of oil yields and quality.

## Introduction

Water is essential for agricultural production and food security. Our freshwater resources are dwindling at an alarming rate. It is estimated that by 2025, around 2 billion people will be affected by absolute water scarcity (Riemenschneider et al., 2016). Thus, growing water scarcity is now one of the leading challenges for sustainable development. This challenge will become more pressing as the world’s population continues to grow, their living standards increase, diets change, and the effects of climate change intensify, increasing temperatures across the world. In this context, more frequent and severe droughts are already having an impact on agricultural production, where rising temperatures translate into increased crop water demand.

Agriculture, which is the most water-demanding economic sector worldwide, is both a major cause and casualty of water scarcity. Farming accounts for almost 70% of all water withdrawals, and up to 95% in some developing countries, with freshwater resources heavily stressed by irrigation and food production (FAO, 2018). In Italy, the dimensions of economic loss in the farming sector were predicting losses of 2 billion Euros (EC, 2018) due to the droughts of summer 2017. In particular, Apulia region (southeast of Italy), which exhibits a Mediterranean climate characterized by hot, dry summer, requires a high volume of irrigation water because many hectares of fruit tree crops (olive, grapes, almond, sweet cherry) account for 80% of the region’s irrigated land (Arborea et al., 2017). Besides, extensive exploitation of wells by Apulian regional farmers is causing the progressive salinization and depletion of relevant portions of the regional aquifers reducing the water available for agriculture (Vivaldi et al., 2019).

Thus, techniques for optimizing water productivity as the regulated deficit irrigation (RDI), where water deficits are imposed during phenological periods when the tree is the least sensitive to water stress, and with little impact on fruit yield (Romero-Trigueros et al., 2017) or the use of non-conventional water sources in agriculture as a component of effective water conservation strategies, are required in regions with water scarcity (Romero-Trigueros et al., 2019). Doing so will not prevent a drought from occurring, but it can help in preventing droughts to result in famine and socioeconomic disruption (FAO, 2018).

With respect to non-conventional water sources, reused reclaimed water (RW) is considered non-expensive and reliable, particularly for irrigation in agriculture. It is estimated that, globally, the market for reuse was on the verge of expansion and expected to outpace desalination in the future. It is foreseen that, by 2030, water reuse will represent about 1.7% (26 billion m^3^ year^−1^) of the total water use (Global Water Market, 2017). RW usually may contain not only essential nutrients, which are beneficial for crop growth and economy of the growers, but also salts, toxic ions, and micropollutants, which discharge into the environment and can accumulate in the soil and crops over time, affecting plants, soils, and underground water bodies (Romero-Trigueros et al., 2014). For this reason, reducing salt concentrations in these water sources, leading to desalinated water, using technologies, could be an imperative need. Nevertheless, the technologies used must be adequately validated in real crops, ensuring their sustainability, and knowing how it affects the fruit yield and quality. Olive crop is considered moderately tolerant to salinity (Gucci and Tattini, 1997; Erel et al., 2019) with water electrical conductivity (EC_w_) between 3 and 6 dS m^−1^, causing no effect to growth or yields (Ayers and Westcot, 1985). In addition, in the Mediterranean region, olive is a major tree crop, and more than 90% of the world’s olive oil is produced. Concretely, in Apulia region, olive is the most representative fruit tree crop irrigated with 383,650 ha cultivated (33 % of total olive orchards in Italy) and with a production of 205,983 t of olive (48% of total Italian production) (ISTAT, 2017; ISMEA, 2018). Salinity tolerance mechanisms of olive trees apparently include a strong ability to exclude potentially toxic ions from above-ground tissues (Kchaou et al., 2010). There are some studies where the saline RW has been used to irrigate olive trees in Mediterranean countries (Greece, Israel, Italy, Spain, Jordan, Egypt, and Tunisia), which reported that the tolerance to salinity depends on the olive varieties. Most of the works evaluated soil properties and leaf nutrients (Aragüés et al., 2005; Ben Rouina et al., 2011; Segal et al., 2011; Petousi et al., 2015; Bourazanis et al., 2016; Erel et al., 2019), root nutrient (Bedbabis et al., 2014), vegetative growth (Kchaou et al., 2010; Ben-Gal et al., 2017), fruit nutrient (Melgar et al., 2009; Batarseh et al., 2011; Bedbabis et al., 2014), and oil yield and quality (Melgar et al., 2009; Ayoub et al., 2016; Tietel et al., 2019).

Regarding the effects of RDI in olive crop, recent works showed that linoleic acid content in olive oil (Hernández et al., 2018), the vegetative growth (Rosecrance et al., 2015; Hernández-Santana et al., 2018), the fruit yield (Gucci et al., 2019) were decreased by water stress. However, a moderate water stress can increase olive oil yield and quality and accelerate fruit maturity (Rosecrance et al., 2015).

To our knowledge, however, nothing has been published about the effects of (i) the irrigation with desalinized water (DW) or of the combination of both water sources (DW and RW) with the RDI strategy and (ii) the strategies on cultivar Arbosana, which is the variety object of study of this work. This cultivar is characterized by early bearing (2nd year after planting), low vigor, and slow canopy growth so that it is the most suitable cultivar to new super high-density olive (SHD) cropping systems (Godini et al., 2011; Rallo and Provenzano, 2013; Vivaldi et al., 2015). SHD olive orchards are spread on over 200,000 ha all over the world on five continents (Olint, 2018). Arbosana, despite being a cultivar widely cultivated has been under-assessed; only Kchaou et al. (2010) studied it in a greenhouse pot experiment with nutrient solution. Indeed, most of works used other olive cultivated varieties, such as Barnea, Leccino (Segal et al., 2011; Ben-Gal et al., 2017; Erel et al., 2019; Tietel et al., 2019), Arbequina (Aragüés et al., 2005; Rosecrance et al., 2015; Hernández et al., 2018; Hernandez-Santana et al., 2018), Chemlali (Ben Rouina et al., 2011; Bedbabis et al., 2014; Bedbabis and Ferrara, 2018), Koroneiki (Petousi et al., 2015; Bourazanis et al., 2016), Nabali Muhassan (Ayoub et al., 2016), Frantoio (Gucci et al., 2019), and Picual (Melgar et al., 2009). 

This work intends to assess the effects of the use of desalinated and saline RW combined with two irrigation strategies, full irrigation (FI) and RDI on (i) fruit yield and ripening indices and (ii) oil yield and quality of olive trees cv Arbosana.

## Materials and Methods

### Experimental Site and Plant Material

The study was conducted at an experimental site located in the southeast of Italy (Bari, Apulia Region) (41°06′41′′N, 16°52′57′′E) (5 m above sea level) during 2017 and 2018. The crop used was 2 years self-rooted olive trees (cv Arbosana) planted on not covered 100-L polyethylene pots (diameter, 50 cm; height, 65 cm). Pots were on the ground with a 1.85 × 2.10 m planting system in rows oriented N-NE to S-SW. The soil texture within the first 90 cm depth was classified as loam (44.78 % sand, 12.32 % clay, and 42.90 % silt) (USDA textural soil classification). 

### Irrigation Treatments

Two irrigation water sources were examined. First was low-cost water DEsalination and SEnsoR Technology (DESERT) DW, obtained by treating secondary wastewater coming from Bari secondary wastewater treatment plant with EC_w_ 1.2 dS m^−1^ by ultrafiltration, active carbon, and reverse osmosis till reaching an EC_w_ of 1.0 dS m^−1^. DESERT is an innovative water desalination and sensor technology compact module for continuously monitoring water quality that has been developed in the framework of the DESERT European project (Water JPI, 2016) with participating partners from Italy, Spain, and Belgium. DESERT technology, to contrast water scarcity and to increase the water quality, enhances the energy savings using solar energy to treat the non-conventional water. The second one is saline RW, which is obtained by mixing the secondary wastewater (EC_w_ 1.2 dS m^−1^) with the brine produced on the DESERT prototype till reaching an EC_w_ of 3 dS m^−1^). 

**Figure 1:**
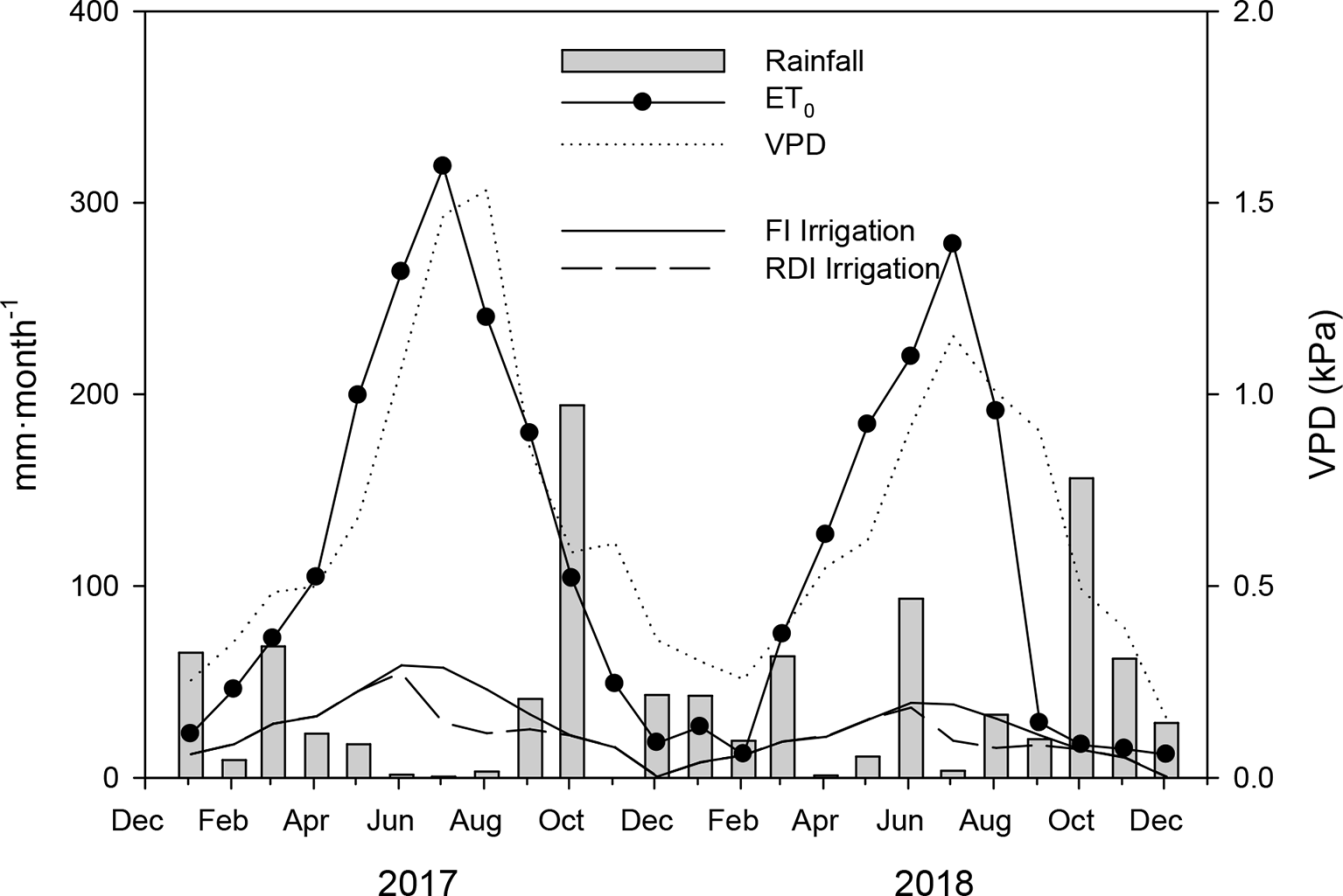
Seasonal evolution of rainfall (mm·month^−1^), reference evapotranspiration (ET_0_, mm·month^−1^), vapor pressure deficit (VPD, kPa), and full (FI) and regulated deficit (RDI) irrigation depths (mm·month^−1^) during 2017 and 2018.

Two irrigation treatments were established for each water source. The first treatment was FI treatment throughout the growing season to fully satisfy crop water requirements (100% ET_c_). The second one was an RDI treatment with an irrigation regime similar to FI, except during the initiation of the first stage of oil accumulation, when it received half the water as applied to the FI (50% ET_c_). This RDI period was chosen because it corresponded to approximately the end of maximum rate of pit hardening and before the rapid phase of fruit growth and oil accumulation begins, thus avoiding the fruit set period (Stage 1), when olive trees are more sensitive to water stress (Gucci et al., 2019; Rosecrance et al., 2015). The DW-RDI was considered as the control treatment. The irrigation was scheduled on the basis of daily evapotranspiration of the crop (ET_c_) accumulated during the previous week. ET_c_ values were estimated by multiplying reference evapotranspiration (ET_0_) (Equation 1) as recommended by FAO (Allen et al., 1998):

(1) $$ET_{c}=Kr\cdot Kc\cdot ET_{0}$$

where Kr is reduction coefficient (Kr = 0.75) and Kc (0.40 Kc_ini_, 0.90 Kc_mid_, 0.65 Kc_end_) is crop coefficient. ET_0_ was calculated by Penman–Monteith methodology, and all data were provided by a climate station located 100 m far from the experimental platform. The monthly evolution of the ET_0_ during the experiment is shown in **Figure 1**. The water was supplied by drip irrigation with three pressure compensated drippers per tree, each with a flow rate of 2 L h^−1^.

All trees received the same amount of NPK macronutrients through a drip irrigation system. Integrated pest management and pruning were those commonly used by growers in the area, and no weeds were allowed to develop within the orchard.

### Water Quality and Plant Water Status

The inorganic solute content, pH and EC_w_ of each irrigation water source were assessed monthly during the irrigation seasons in 2017 and 2018. The samples were collected in glass bottles, transported in an ice chest to the laboratory, and stored at 5°C before being processed for chemical and physical analyses. The concentrations of macronutrients (N, K, P, Ca, and Mg) and micronutrients including B were determined by inductively coupled plasma optical emission spectrometer (ICP-ICAP 6500 DUO Thermo, England). Anions (Cl^−^, NO_3_^−^, PO_4_^3−^, and SO_4_^2−^) were analyzed by ion chromatography with a liquid chromatograph (Metrohm, Switzerland). EC_w_ was determined using a PC-2700 meter (Eutech Instruments, Singapore), and pH was measured with a pH-meter Crison-507 (Crison Instruments S.A., Barcelona, Spain).

Stem water potential (SWP) was determined weekly during the irrigation periods at midday, using a pressure chamber (model 3000, Soil Moisture Equipment Corp., California, USA), according to Scholander et al. (1965), on one fully expanded leaf per tree from the mid-shoot area, which were bagged within foil-covered aluminum envelopes at least 2 h before the measurement (Shackel et al., 1997).

### Fruit Yield and Ripening Indices

The harvesting was made on October 31, 2017, and November 21, 2018, at the appropriate ripening stage, when detachment index reached at least 2 N g^−1^. All olives were manually and separately collected and weighed to determine the fruit yield (g tree^−1^). The fruit number per tree was calculated by dividing the fruit yield by average single fruit weight.

Fifty olives from each tree of each treatment were randomly sampled immediately after harvest to determine the different fruit ripening indices, following the methodology reported by Camposeo et al. (2013). For fresh weight (FWe), dry weight (DWe), and water content (WC) calculated as (FWe-DWe)·FWe−1·100, fruits were brought to the laboratory to determine the FWe on a digital balance (XS105 Dual Range, Mettler Toledo, Greifensee, Switzerland). Then, these were oven-dried for 48 h at 65°C, cooled for 30 min in a desiccator and again weighted. The detachment index (DI; N g^−1^) was calculated as: DI = DF·FWe^−1^ where DF is the detachment force (N) measured using a manual dynamometer (Somfy Tec). Fruit firmness was measured with a penetrometer ADEMVA (mod.TR) using a tip Ø 2 mm on the equatorial zone. 

Fruit color was determined as pigmentation index (PI) calculated in Equation (2):

(2) $$PI=\sum_{i=0}^{5}\frac{i\times ni}{N}$$

where i is the number of the group, ni is the number of fruits per group, N is the total number of fruits in the sample. The procedure consisted in distributing the sample of olives in six groups, according to the following characteristics: group 0, green skin; group 1, <50% black skin with white flesh; group 2, ≥50% black skin with white flesh; group 3, 100% black skin with white flesh; group 4, 100% black skin with <50% purple flesh; and group 5, 100% black skin and ≥50% purple flesh (0 ≤ PI ≤ 5).

### Oil Extraction and Quality

Fatty yield (%), humidity (%), and acidity (%) were determined by near-infrared spectroscopy in part of fruits which were crushed in a hammer mill (FOSS Olivia™, Barcelona, Spain), the resulting olive paste malaxed at 25°C for 30 min (Servili et al., 2007). 

To determine oil industrial extractability (%) after olive paste was obtained, the oil was extracted and separated by vertical centrifugation, collected, and left to decant. The oil samples were filtered and stored at 14°C in a dark and cool place in amber glass bottles until analysis. Results were also expressed as oil content per dry and fresh fruit weight (%) and oil yield (g_oil_ tree).

Olive oil free acidity (FA), given as percent of oleic acid (C18:1), (% C18:1 per 100 g olive oil), peroxide value (meq O_2_ kg^−1^ oil), and UV determinations (K_232_, K_270_, and ΔK) were carried out according to the European Union Commission Reg. 61/2011 (EEC, 2011) and International Olive Council (IOC) standard methods. The parameters or extinction coefficients, K_232 _and K_270_, have oil absorbance at 232 and 270 nm, respectively, and ΔK was calculated from the absorbances at 232, 268, and 274 nm. Spectrophotometric determinations, K_232_, K_270_, and ΔK analyses, were carried out using a Shimadzu UV-1601 spectrophotometer (Shimadzu, Kyoto, Japan).

Profiles of fatty acids methyl esters (FAME) were determined by gas chromatography (EEC, 2011). Olive oil was diluted in hexane (1% oil) and 0.4-ml solution was added to 0.2-ml methanol solution with KOH 2 N. The mixture was vigorously shaken for 1 min and 2 µL of the hexane organic phase was collected for the GC injection. A Shimadzu mod. GC-17A equipped with flame ionization detected (FID) (Shimadzu Italia, Milan, Italy) was used for the analysis. The acquisition software was Class-VP Chromatography data system 4.6 (Shimadzu Italia, Milano, Italy). A FAME capillary column, 60 m, 0.25 mm i.d. with 0.25 mm 50% cyanopropyl-methyl phenyl silicone, was used (Quadrex Corporation, New Heave, USA). Chamber was held at 170°C for 20 min using a rate of 10°C min^−1^ until 220°C, held for 5 min. Injector temperature and FID temperature was 250°C; carrier gas, Helium; column flow, 2 ml min^−1^; split ratio, 1/60; injected volume, 20 µl. Peaks identification was performed by comparing retention times of fatty acids with those of pure compounds (mixture of pure methyl esters of fatty acids; Larodan, Malmoe, Sweden) injected in the same condition. 

Phenolic compounds were extracted and determined according to Caponio et al. (2015) with slight modifications. Extraction was carried out on 1 g of oil by adding 1 ml of hexane and 5 ml of methanol/water (60:40v/v). After vortexing for 1 min and centrifuging at 4000 rpm for 10 min, the hydroalcoholic phase was recovered and filtered through nylon filters (pore size 0.45 μm, Sigma-Aldrich, Milan, Italy). Then, 100 ml of extract were mixed with 100 ml of Folin–Ciocalteu reagent by Folin and Ciocalteau (1927) and after 4 min, with 800 ml of a 5% (w/v) solution of sodium carbonate. The mixture was stored in the dark for 30 min, and the total phenol content was determined at 750 nm by a Shimadzu UV-1601 spectrophotometer (Shimadzu, Kyoto, Japan). The total phenolic content was expressed as gallic acid equivalents (mg·kg^−1^).

Chlorophyll and carotenoids determination was carried out by measuring the absorption of the oil/hexane solution (1:1 v/v) at wavelengths of 670 nm for chlorophyll and 450 nm for carotenoids using a Shimadzu UV-1601 spectrophotometer (Shimadzu, Kyoto, Japan) (Minguez et al., 1991).

### Experimental Design and Statistical Analysis

A total of 40 trees were used in this study (10 per treatment). The experimental design of each irrigation treatment was five replicates distributed following a completely randomized design. Each replica consisted of two trees. To evaluate the fruit yield and ripening indices, all trees per treatment (10 trees per treatment) were used. For the study of the SWP, oil yield and quality five trees per treatment (one per replicate) were evaluated.

A weighted analysis of variance (ANOVA) followed by Tukey’s test (*P* ≤ 0.05) was used for assessing differences among treatments. Linear regressions among the different variables measured were calculated. Pearson’s correlation coefficients were used to assess the significance of these relationships. These statistical analyses were performed using SPSS (vers. 23.0 for Windows, SPSS Inc., Chicago, IL). To discriminate significant differences among parameters of different linear regressions (slope and intercept) analysis of covariance (ANCOVA) were performed using Statgraphics software (Statgraphics Plus for Windows Version 4.1). The data also were analyzed using a two-way ANOVA with water quality and water amount as the main factors.

## Results

### Climatic Data

Climate conditions at the experimental site followed typical Mediterranean patterns, with hot and dry weather from May to September, reaching ET_0_ values of 318.81 and 278.28 mm·month^−1^ in 2017 and 2018, respectively, and being mild and wet for the rest of the year. The total rainfall was 467 and 535 mm in 2017 and 2018, respectively. Most of the annual rainfall occurred between September and May (**Figure 1**). The daily average vapor pressure deficit (VPD) values reached 1.53 and 1.15 kPa in August of 2017 and 2018, respectively (**Figure 1**). There was a clear different climatic pattern between years. The first year was hotter and more arid than the second one. In particular, during the ripening period (from September to November), the sum of ET0 values was much higher in 2017 than in 2018 (332.5 mm vs 60.6 mm, respectively).

### Irrigation Water Quality and Plant Water Status

The results of the chemical analysis of both irrigation water sources used in the experiment, DW and RW, during 2017 and 2018, are presented in **Table 1**. In general, both waters were characterized by a slight alkaline; within the range of proper irrigation water (Bedbabis et al., 2010). The pH values were weakly higher in RW than in DW, and both within the limits allowed by the D.L. 185/2003. RW had significantly higher salinity than DW (31 ds·m^−1^ versus 1 ds·m^−1^). The sodium absorption ratio (SAR) also was higher in RW than DW, and both sources presented values below the limit. The Cl^−^ concentrations were around 200 mg·L^−1 ^for DW and almost double (380 mg·L^−1^) for RW, exceeding, in this last case, the threshold values indicated in D.L. 185/2003. Likewise, Na presented higher levels in the RW than in the DW, mainly in 2017. Regarding nutrients and elements considered essential for plant growth and development, RW contained higher amounts of NO_3_^−^, PO_4_^3−^, and K, with respect to DW. Similarly, Mg and Ca concentrations in RW were around the double in comparison with DW. Boron, an important micronutrient for olive tree production (Saadati et al., 2013) was found in both sources at a quite similar concentration. The micronutrients as Fe, Mn, and Zn also were slightly higher in RW than in DW (Fe, only in 2018, and Mn presented values about almost double both years). Others toxic heavy metals as Cd, Cr, and Pb were not detected in any of the water sources, whereas the highest Al and Ni levels were in RW.

**Table 1:** Physical and chemical properties for DESERT desalinated water (DW) and reclaimed water (RW) in 2017 and 2018.

Property	Units	2017		2018		Limits D.L. 185/2003
		DW	RW	DW	RW	
pH		7.53 ± 0.31	8.15 ± 0.20	8.11 ± 0.32	8.44 ± 0.34	6–9.5
EC_w_	dS·m^−1^	1.00 ± 0.15	3.00 ± 0.45	1.13 ± 0.61	3.00 ± 0.89	3
SAR		3.7 ± 0.42	7.2 ±1.52	4.79 ± 1.94	5.69 ± 1.62	10
Ca	mg L^−1^	56.28 ± 11.30	121.3 ± 22.1	50.76 ± 21.52	108.05 ± 57.15	–
Mg	mg L^−1^	20.9 ± 5.40	35.5 ± 6.10	18.31 ± 8.12	35.96 ± 16.82	–
K	mg L^−1^	20.67 ± 8.81	42.76 ± 6.30	20.37 ± 9.77	33.54 ± 12.60	–
Na	mg L^−1^	148.4 ± 53.2	353.2 ± 48.7	160.10 ± 85.67	270.66 ± 126.36	–
B	mg L^−1^	0.14 ± 0.06	0.15 ± 0.07	0.13 ± 0.05	0.14 ± 0.04	1.00
Mn	mg L^−1^	0.08 ± 0.01	0.16 ± 0.02	0.09 ± 0.03	0.17 ± 0.06	–
Zn	mg L^−1^	0.03 ± 0.00	0.05 ± 0.01	0.03 ± 0.02	0.04 ± 0.01	
Fe	mg L^−1^	0.04 ± 0.01	0.04 ± 0.01	0.07 ± 0.06	0.13 ± 0.12	–
Cu	mg L^−1^	0.000 ± 0.000	0.009 ± 0.004	0.009 ± 0.005	0.015 ± 0.001	
Al	mg L^−1^	0.04 ± 0.01	0.05 ± 0.02	0.06 ± 0.03	0.09 ± 0.06	
Ni	mg L^−1^	0.012 ± 0.000	0.026 ± 0.001	0.022 ± 0.023	0.114 ± 0.176	
NO_3_^−^	mg L^−1^	15.83 ± 2.53	36.16 ± 9.28	28.39 ± 25.08	42.70 ± 19.93	–
PO_4_^3−^	mg L^−1^	1.3 ± 0.61	3.1 ± 0.52	2.01 ± 0.52	2.51 ± 1.45	2
SO_4_^−2^	mg L^−1^	97.98 ± 16.2	227.4 ± 37.5	92.37 ± 66.11	144.92 ± 92.15	500
F^−^	mg L^−1^	0.22 ± 0.09	0.38 ± 0.11	0.22 ± 0.12	0.30 ± 0.17	–
Cl^−^	mg L^−1^	198.1 ± 54.1	379.5 ± 72.3	199.77 ± 184.71	380.18 ± 181.33	250

Values are averages ± SD of 12 individual samples taken throughout the crop cycle. EC_w_, water electrical conductivity; RW, reclaimed water; DW, DESERT desalinized water.

The irrigation season lasted from May 1 to October 31 and from May 15 to November 9 for 2017 and 2018, respectively. The amounts of water applied were 3679.63 and 3062.34 m^3^·ha^−1 ^for FI and RDI treatments in 2017 and 2460.49 and 2011.23 m^3^·ha^−1 ^for FI and RDI treatments in 2018, respectively (**Figure 1**). Therefore, the RDI treatment saved about 21% of irrigation water. The RDI period in 2017 began on DOY 180 (29^th^ June 2017) and ended on 213 (August 1, 2017). The RDI period in 2018 started on DOY 180 (June 29, 2018) and ended on DOY 243 (August 31, 2018). The RDI period in 2017 lasted 1 month less than in 2018 for two reasons: (i) the trees were very young and (ii) the trees were shortly transplanted (1 year).

The water quality did not affect the SWP. However, the RDI treatments reached SWP values significantly more negative (−1.90 and −3.05 MPa in 2017 and 2018, respectively) than the control treatment (−1.02 and −1.29 MPa in 2017 and 2018, respectively) during the RDI periods. The SWP was lower in the water stressed trees during the second year than in the first one because the RDI period was longer.

### Properties of the Fruit Production

**Figure 2:**
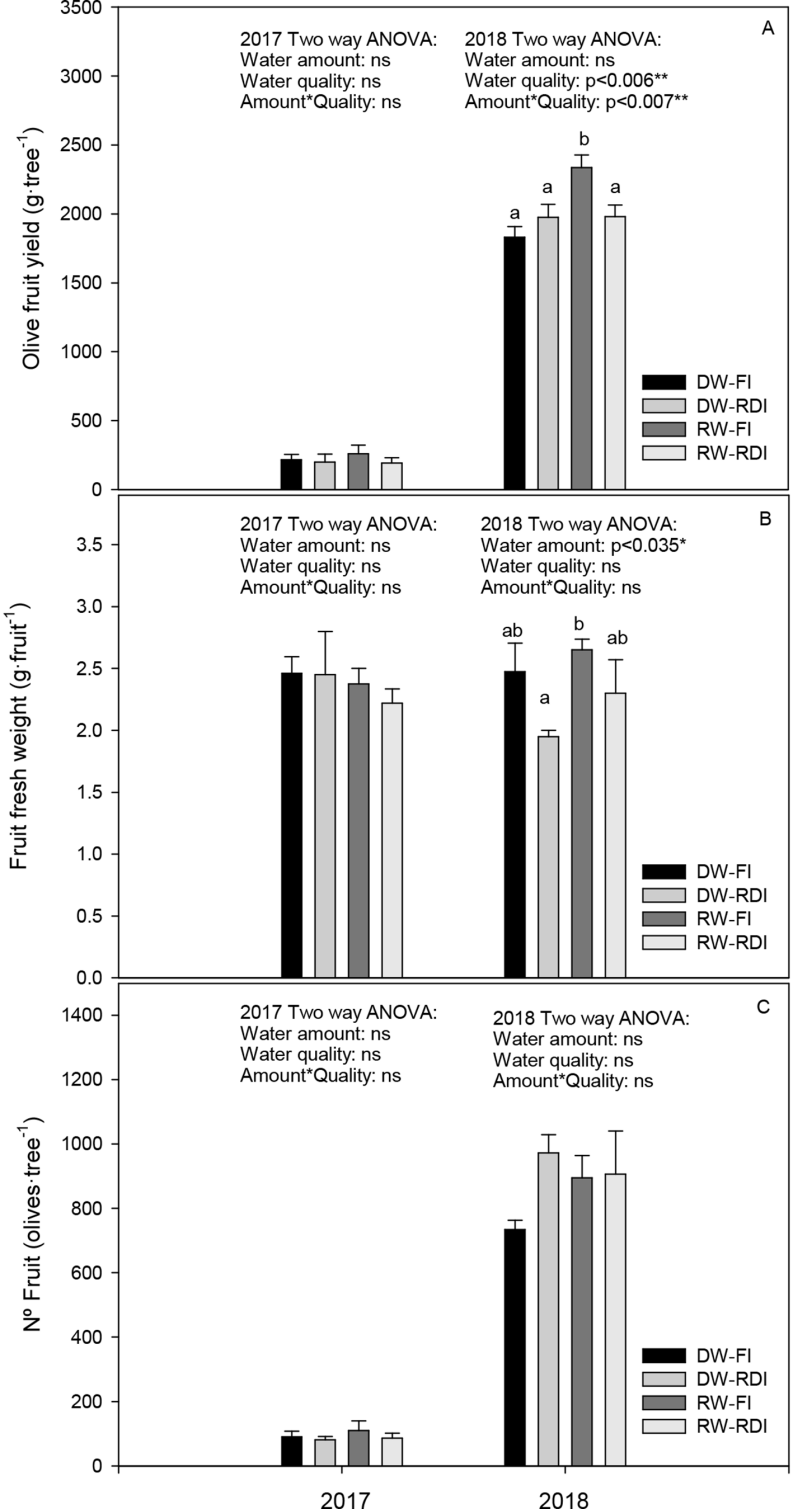
**(A)** Olive fruit yield (g tree−1), **(B)** fruit fresh weight (g fruit−1) and **(C)** number of fruit (olives tree−1) for each treatment: DW-FI (DESERT water-full irrigation), DW-RDI (DESERT water-regulated deficit irrigation), RW-FI (reclaimed water-full irrigation), and RW-RDI (reclaimed water-regulated deficit irrigation) for 2017 and 2018.

Harvest data for each year are shown in **Figure 2**. In general, the fruit yield was very low in the first year of the experiment with an average value of 218 g per tree. In the following year, Arbosana trees started to bearing, with a yield of 2.01 kg per tree. The average individual fruit weight ranged between 2.2 and 2.5 g and between 2.0 and 2.6 g for 2017 and 2018, respectively. The fruit number per tree ranged from 81.3 to 110.3 in 2017 and from 734.3 to 972.0 in 2018. Considering the different treatments, there were no differences among them neither in yield, nor in fruit weight or number of fruit in 2017. However, the RW-FI treatment had a fruit yield significantly higher than the rest of treatments, about 35% more than DW-FI (**Figure 2A**) in 2018. This was mainly due to an increase in the olive weight (7%) and to a greater number of fruits per tree (21%), although there were no significant differences in these two parameters (**Figures 2B, C**). Water stress caused by the RDI strategies did not affect significantly the fruit yield, regardless of the water quality, although we observed an increase in the number of fruits per tree (32.4% and 23.5% for DW-RDI and RW-RDI, respectively) and a decrease in the fruit weight (21% and 6.8% for DW-RDI and RW-RDI, respectively) with respect to the DW-FI, mainly in the DW-RDI.

Regarding fruit ripening indices at harvest (**Table 2**), neither quality water (RW treatments) nor water amount (RDI treatments) affected significantly detachment index, fruit firmness, and PI. Nevertheless, some trends were observed. Fruits from RW trees (i) were detached more easily than control trees, although the fruit detachment force was not significantly lower in the RW-FI versus the DW-FI (**Table 2**), and (ii) had a higher PI than the DW-FI in the first year (63%). Besides, in 2017, the mean PI was 1.5 and in the second one decreased about 0.9. During the RDI period, DW-RDI fruits looked wrinkled. When such period ended, fruits visually recovered the firmness; although the lowest values of firmness were observed in this treatment at harvest. Finally, the fruit WC was significantly higher in the RDI treatments in 2018 (4.9% and 4.2% for DW-RDI and RW-RDI, respectively).

**Table 2:** Ripening indices: water content, detachment index, fruit firmness and pigmentation index for each treatment: DW-FI (DESERT water-full irrigation), DW-RDI (DESERT water-regulated deficit irrigation), RW-FI (reclaimed water-full irrigation) and RW-RDI (reclaimed water-regulated deficit irrigation) in 2017 and 2018.

	Water content (%)		Detachment Index (N·g^−1^)		Fruit firmness (N)		Pigmentation Index	
	2017	2018	2017	2018	2017	2018	2017	2018
DW-FI	58.2 ± 0.7a	56.9 ± 0.7a	2.28 ± 0.12a	2.23 ± 0.15a	6.54 ± 0.55a	7.68 ± 0.43a	1.32 ± 0.24a	0.84±0.29a
DW-RDI	59.3 ± 1.9a	59.6 ± 0.3b	2.25 ± 0.25a	2.25 ± 0.24a	5.50 ± 0.40a	7.57 ± 0.14a	1.25 ± 0.04a	0.93±0.17a
RW-FI	58.0 ± 1.2a	58.4 ± 0.1ab	2.13 ± 0.13a	2.10 ± 0.13a	5.75 ± 0.47a	7.70 ± 0.29a	2.15 ± 0.25a	0.80±0.27a
RW-RDI	59.9 ± 0.5a	59.2 ± 0.4b	2.42 ± 0.17a	2.23 ± 0.22a	6.02 ± 0.17a	7.73 ± 0.14a	1.34 ± 0.24a	0.88±0.26a
Water amount	ns	*p* < 0.001***	ns	ns	ns	ns	ns	ns
Water quality	ns	ns	ns	ns	ns	ns	ns	ns
Amount*quality	ns	p < 0.041*	ns	ns	ns	ns	ns	ns

Different letters within the same column indicate significant differences among treatments according to Tukey’s Test (p < 0.005).

ns, not significant.

### Oil Determination

**Figure 3:**
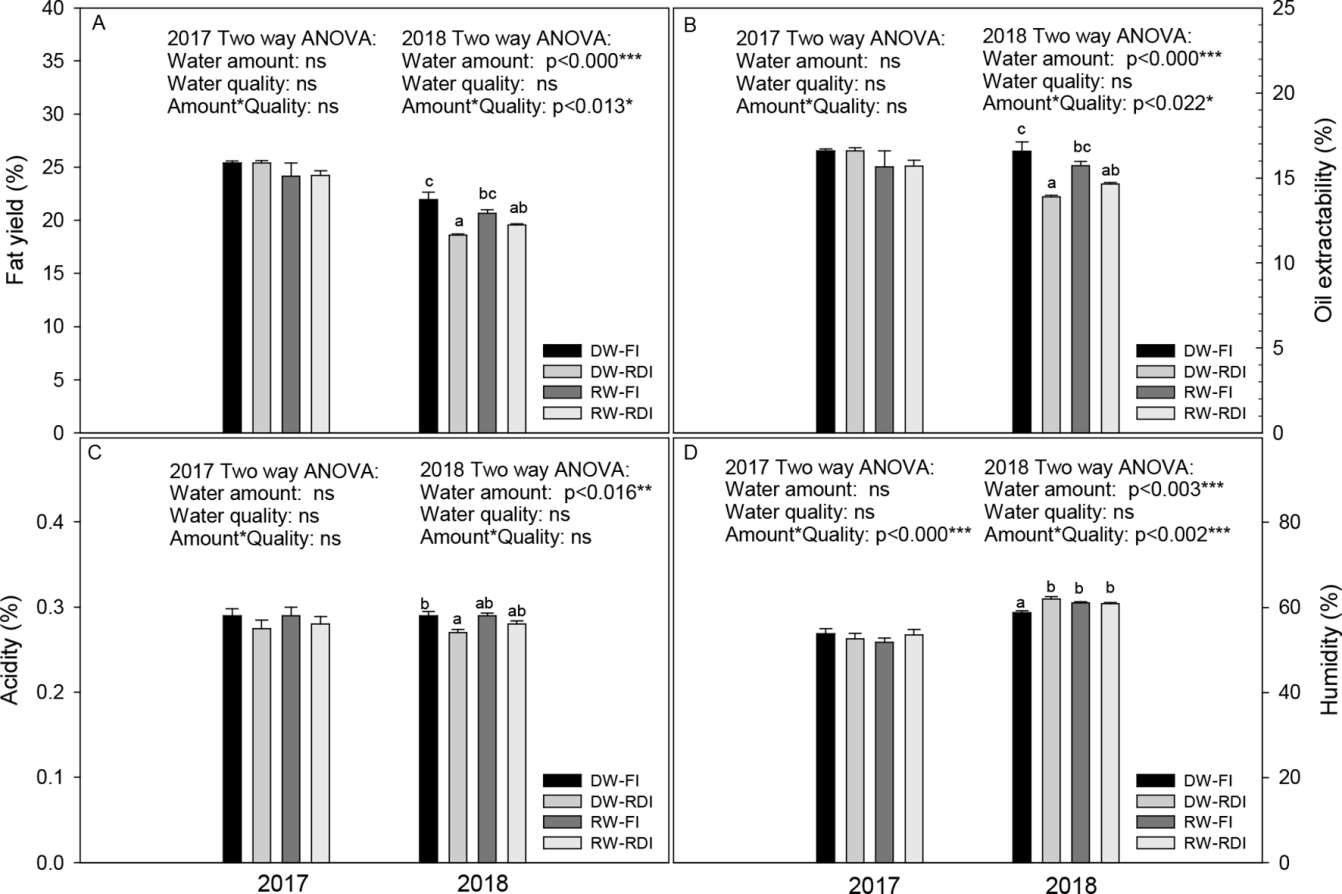
**(A)** Fatty yield (%), **(B)** oil extractability (%), **(C)** acidity (%) and **(D)** humidity (%) for each treatment: DW-FI (DESERT water-full irrigation), DW-RDI (DESERT water-regulated deficit irrigation), RW-FI (reclaimed water-full irrigation), and RW-RDI (reclaimed water-regulated deficit irrigation) in 2017 and 2018.

The fatty yield was affected by water stress in 2018 because both RDI treatments had less fatty performance than the DW-FI (a reduction of 15.2 and 11.0% for DW-RDI and RW-RDI, respectively) (**Figure 3A**). Similarly, oil industrial extractability also was decreased by RDI strategies in 2018 (a reduction of 16.2% and 11.6% for DW-RDI and RW-RDI, respectively) (**Figure 3B**). Humidity percentage in olive paste was higher in all stressed treatments compared with DW-FI in the second year. With respect to acidity, olive paste of RDI treatments also presented a mild reduction in its levels in both years, being significant for the DW-RDI in 2018 (6.1%) (**Figure 3C**). Moreover, taking into account all the treatments, humidity was significantly correlated to fatty yield (*R* = 0.82, *p* < 0.005***) and oil extractability (*R* = 0.80, *p* < 0.005***), so that as humidity increased, the fatty yield and oil extractability decreased. With less significance, humidity also correlated negatively with acidity (*R* = 0.60, *p* < 0.01**). In this sense, overall, the levels of fatty yield and oil extractability decreased by 4% and 1.2%, respectively, from 2017 to 2018. The fatty yield average values were around 24% in 2017 and decreased about 20% in 2018 and, like the oil extractability averages, were 16.4% in 2017 and 15.2% in 2018. On the contrary, humidity was higher in 2018 (61%) than 2017 (53%).

**Figure 4:**
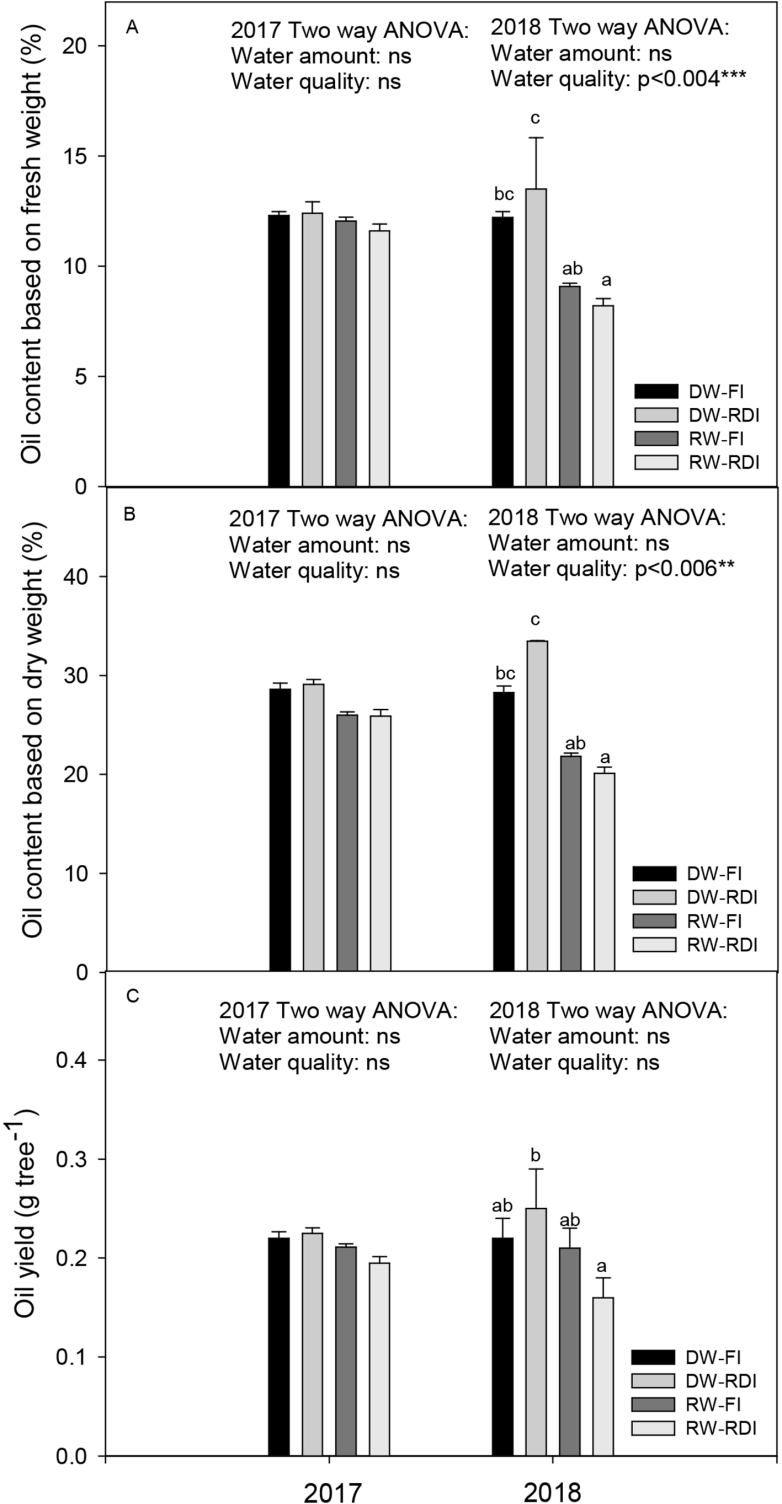
Oil content based on **(A)** fresh fruit weight (%), **(B)** oil content based on dry fruit weight (%) and **(C)** oil yield (g tree−1) for each treatment: DW-FI (DESERT water-full irrigation), DW-RDI (DESERT water-regulated deficit irrigation), RW-FI (reclaimed water-full irrigation) and RW-RDI (reclaimed water-regulated deficit irrigation) in 2017 and 2018.

There were no significant differences among treatments for the oil content per fresh and dry fruit weight and for the oil yield in the first year of the experiment. However, oil content was affected by the water quality in the next year by two-way ANOVA (**Figures 4A, B**). Concretely, the oil content based on fresh fruit weight ranged from 8.20% to 13.50% and in the RW-FI and RW-RDI a reduction by 25% and 33%, respectively, was found (**Figure 4A**). In DW-RDI, on the contrary, a tendency to increase (10.6%) was observed. Oil content based on dry fruit weight showed the same behavior than oil content based on fresh weight in 2018, with values between 20% and 33%. A decrease by 22.9% in RW-FI and also a strong decrease by 39.93% in the RW-RDI, with respect to the DW-FI, were observed. Contrary, we observed a slight increase by 18.3% in the DW-RDI (**Figure 4B**).The oil yield was 0.22, 0.25, 0.21, and 0.16 g_oil_ tree^−1^ for DW-FI, DW-RDI, RW-FI, and RW-RDI, respectively, in 2018. Despite the two-way ANOVA indicating that this parameter was not affected by the quality or quantity of water (**Figure 4C**), Tukey’s test did show a tendency, that is, the oil yield decreased by 1.72% in RW-FI, despite such treatment having the highest olive fruit production, and markedly decreased by 24.8% in trees with the combination RWRDI. An increase by 14.5% in the DW-RDI was also found.

### Oil Quality

**Figure 5:**
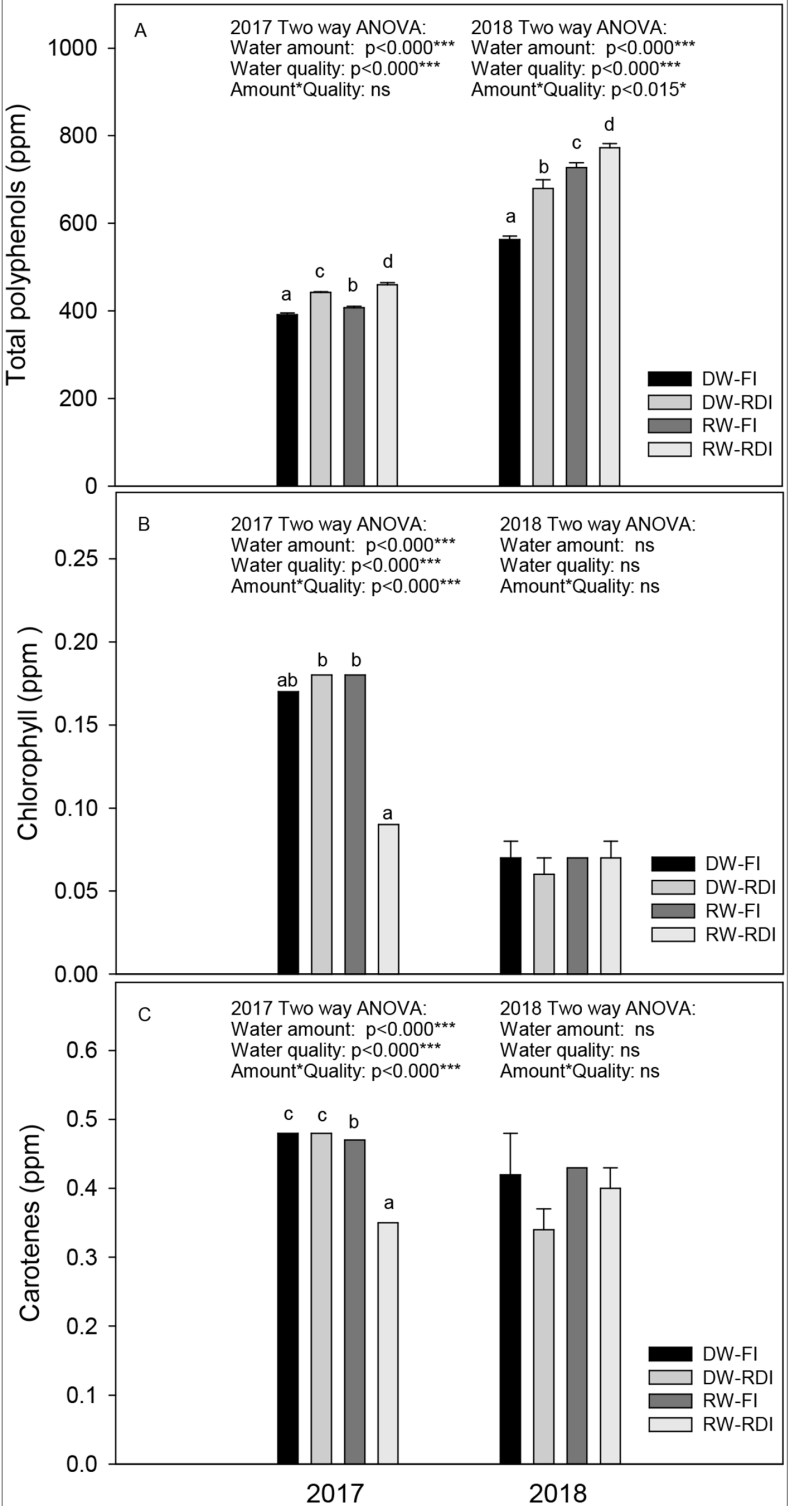
**(A)** Total polyphenols, **(B)** chlorophyll and **(C)** carotenes (ppm) in oil samples of each treatment: DW-FI (DESERT water-full irrigation), DW-RDI (DESERT water-regulated deficit irrigation), RW-FI (reclaimed water-full irrigation), and RW-RDI (reclaimed water-regulated deficit irrigation) in 2017 and 2018.

The most nutritional and chemical quality parameters accepted in oil evaluation were evaluated (**Tables 3** and **4**, and **Figure 5**).

**Table 3:** Oil quality chemical parameters: free acidity (FA), peroxides, K_232_, K_270_ and ΔK for each treatment: DW-FI (DESERT water-full irrigation), DW-RDI (DESERT water-regulated deficit irrigation), RW-FI (reclaimed water-full irrigation) and RW-RDI (reclaimed water-regulated deficit irrigation) in 2017 and 2018.

		DW-FI	DW-RDI	RW-FI	RW-RDI	Water amount	Water quality	Amount *Quality
FA (%)	2017	0.23 ± 0.01ab	0.20 ± 0.01a	0.23 ± 0.01ab	0.25 ± 0.01b	ns	**	*
	2018	0.33 ± 0.06b	0.28 ± 0.03ab	0.25 ± 0.03ab	0.20 ± 0.00a	ns	ns	ns
Peroxides (meq O_2_·kg^−1^)	2017	7.80 ± 0.01b	7.64 ± 0.05a	8.52 ± 0.01c	8.81 ± 0.01d	*	***	***
	2018	10.93 ± 0.13c	10.00 ± 0.09ab	10.33 ± 0.18bc	9.60 ± 0.17a	***	***	ns
K_232_	2017	1.55 ± 0.00a	1.62 ± 0.00c	1.54 ± 0.00a	1.58 ± 0.00b	***	***	***
	2018	2.29 ± 0.05a	2.31 ± 0.08a	2.18 ± 0.13a	2.41 ± 0.03a	ns	ns	ns
K_270_	2017	0.14 ± 0.00b	0.18 ± 0.00c	0.16 ± 0.00b	0.12 ± 0.00a	ns	***	***
	2018	0.17 ± 0.01a	0.18 ± 0.02a	0.16 ± 0.01a	0.15 ± 0.02a	ns	ns	ns
ΔK	2017	−0.004 ± 0.000b	−0.005 ± 0.000a	−0.004 ± 0.000c	−0.003 ± 0.000d	ns	***	***
	2018	−0.004 ± 0.001a	−0.002 ± 0.000a	−0.005 ± 0.001a	−0.004 ± 0.001a	ns	ns	ns

Different letters within the same row indicate significant differences among treatments for the different parameters and years according to Tukey’s test (*p < 0.005 ; **p < 0.01; ***p < 0.005).

ns, not significant.

**Table 4:** Profiles of fatty acids methyl esters in the oil samples of each treatment: DW-FI (DESERT water-full irrigation), DW-RDI (DESERT water-regulated deficit irrigation), RW-FI (reclaimed water-full irrigation) and RW-RDI (reclaimed water-regulated deficit irrigation) in 2017 and 2018.

Parameter		DW-FI	DW-RDI	RW-FI	RW-RDI	Water amount	Water quality	Amount *quality
C16:0 (%)	2017	15.34 ± 0.00a	15.37 ± 0.01a	15.66 ± 0.01b	15.71 ± 0.01c	***	***	ns
	2018	15.69 ± 0.02a	15.74 ± 0.12a	15.55 ± 0.09a	15.70 ± 0.07a	ns	ns	ns
C16:1 (%)	2017	1.81 ± 0.01b	1.72 ± 0.01a	1.78 ± 0.01b	1.87 ± 0.01c	ns	***	***
	2018	1.68 ± 0.03a	1.72 ± 0.01ab	1.68 ± 0.02a	1.79 ± 0.03b	*	ns	ns
C17:0 (%)	2017	0.11 ± 0.00a	0.11 ± 0.00a	0.11 ± 0.00a	0.13 ± 0.00a	ns	ns	ns
	2018	0.12 ± 0.01a	0.11 ± 0.00a	0.12 ± 0.00a	0.11 ± 0.01a	ns	ns	ns
C17:1 (%)	2017	0.27 ± 0.00b	0.36 ± 0.00c	0.26 ± 0.00b	0.25 ± 0.00a	***	***	***
	2018	0.27 ± 0.01ab	0.35 ± 0.00c	0.24 ± 0.01a	0.28 ± 0.01b	***	***	***
C18:0 (%)	2017	2.12 ± 0.01c	2.11 ± 0.01c	2.08 ± 0.01b	2.03 ± 0.02a	*	***	ns
	2018	2.17 ± 0.02b	2.15 ± 0.04ab	2.07 ± 0.03a	2.11 ± 0.02ab	ns	*	ns
C18:1 (%)	2017	70.48 ± 0.02c	70.45 ± 0.01c	69.76 ± 0.13b	69.42 ± 0.06a	*	***	ns
	2018	70.17 ± 0.13bc	70.57 ± 0.14c	69.56 ± 0.29ab	69.03 ± 0.15a	ns	***	*
C18:2 (%)	2017	8.43 ± 0.01b	8.33 ± 0.01a	8.93 ± 0.02c	9.10 ± 0.00d	*	***	***
	2018	8.48 ± 0.05a	8.24 ± 0.03a	9.26 ± 0.21b	9.16 ± 0.16b	ns	***	ns
C18:3 (%)	2017	0.67 ± 0.01a	0.66 ± 0.01a	0.63 ± 0.01a	0.67 ± 0.01a	ns	ns	*
	2018	0.65 ± 0.01a	0.66 ± 0.01a	0.65 ± 0.01a	0.64 ± 0.01a	ns	ns	ns
C20:0 (%)	2017	0.36 ± 0.01a	0.41 ± 0.01b	0.44 ± 0.01c	0.39 ± 0.01b	ns	**	***
	2018	0.42 ± 0.01ab	0.42 ± 0.01ab	0.40 ± 0.00a	0.43 ± 0.01c	*	ns	ns
C20:1 (%)	2017	0.33 ± 0.01b	0.27 ± 0.01a	0.32 ± 0.00b	0.36 ± 0.01b	ns	***	***
	2018	0.28 ± 0.01a	0.33 ± 0.01a	0.34 ± 0.01a	0.27 ± 0.03a	ns	ns	**
C22:0 (%)	2017	0.12 ± 0.00a	0.15 ± 0.00c	0.14 ± 0.00b	0.13 ± 0.00ab	***	ns	***
	2018	0.15 ± 0.01a	0.13 ± 0.00a	0.25 ± 0.01b	0.18 ± 0.03a	**	***	ns
C24:0 (%)	2017	0.066 ± 0.001a	0.075 ± 0.000b	0.060 ± 0.001a	0.061 ± 0.003a	ns	***	*
	2018	0.072 ± 0.002a	0.063 ± 0.003a	0.060 ± 0.002a	0.061 ± 0.001a	ns	ns	ns
C18:2/C18:3 ratio	2017	12.62 ± 0.12a	12.71 ± 0.27a	14.25 ± 0.17b	13.65 ± 0.16b	ns	***	ns
	2018	12.95 ± 0.26a	12.41 ± 0.12a	14.21 ± 0.58b	14.28 ± 0.16b	ns	***	ns

Different letters within the same row indicate significant differences among treatments for the different parameters and year according to Tukey’s Test (*p < 0.005 ; **p < 0.01; ***p < 0.005).

ns, not significant.

Taking into account the different irrigation treatments, the FA had no clear tendency (**Table 3**). We observed that it slightly decreased in DW-RDI in 2 years, similar to the data found for the acidity during industrial extraction (**Figure 3**). The peroxides decreased in DW-RDI in both years and increased in RW treatments in 2017, with respect to the control trees. The RW-RDI did not show a clear tendency for both years. The K_232_ and K_270_ indices are also indicators of the presence of oxidation compounds in oil, other than peroxides. The mean values of the specific extinction coefficients ranged from 1.51 to 2.41 and from 0.12 to 0.18, respectively, for K_232_ and K_270_. K_232_ had the highest values in the RDI treatments, mainly in DW-RDI, but was only significant in 2017. As for K_270_, it increased in the DW-RDI and decreased in the RW-RDI, being statistically significant only in 2017. The delta K index showed the highest values in RW treatments in 2017. In general, it is also important to highlight, that in the second year, the values of peroxides and K_232_ were higher than in the first year in all cases.

On the other hand, significant effects among treatments on the fatty acid (FAME) composition of the major fraction of the olive oil, also known as saponifiable fraction, were observed (**Table 4**). The saturated and monounsaturated acids palmitic (C16:0) and palmitoleic (C16:1) were affected by quality and amount of water. C16:0 increased by RW (FI and RDI) only in 2017 and C16:1 by the combination of water and saline stress (RW-RDI). The saturated stearic acid (C18:0) and monounsaturated oleic acid (C18:1) significantly decreased in the RW treatments, mainly in the RW-RDI, this last one ranging between 69.03% and 70.57%. The polyunsaturated linoleic (C18:2), known as ω6, increased by RW around 9.2% and 7.1% in 2017 and 2018, respectively, being more marked again in the RW-RDI for 2017. However, linolenic acid (C18:3, ω3) was not affected by any treatment, ranging between 0.63% and 0.67%. Consistently, the C18:2/C18:3 ratio increased about 10.3% in both RW treatments (FI and RDI). As for the acids with hydrocarbon chain of 20 or more carbons, C20:0 was higher in all treatments, with respect to the DW-FI in 2017 and only in the RW-RDI in the next year. The acid C20:1 decreased by RDI (DW-RDI) in 2017. C22:0 obtained the higher values in the DW-RDI and the RW-FI. Finally, C24:0 increased in the DW-RDI and tended to decrease by effect of RW in the first year. 

The main FAME of olive oil was oleic acid consisting around 70% of the FAMEs found (**Table 4**). The second most abundant FAME was palmitic, and the third one was the polyunsaturated FAME linoleic. The order from higher to lower concentration of the different acids was as follows: 

C18:1>C16:0>C18:2>C18:0>C16:1>C18:3>C20:0>C20:1>C17:1>C22:0>C17:0>C24:0

Within the unsaponifiable minority fraction of olive oil, polyphenols and pigments were evaluated (**Figure 5**). Total polyphenols content ranged between 390 and 460 ppm and between 562 and 772 ppm in the first and second year, respectively. Their levels significantly improved in all treatments, with respect to the control trees, mainly in RW-RDI. The increases were 12.9%, 4.0%, and 17.5% in 2017 and 20.9%, 29.3%, and 37.3% in 2018 for DW-RDI, RW-FI, and RW-RDI, respectively. The differences among treatments were higher in 2018 than in 2017. This response can be explained by the highest values of 2018 (**Figure 5A**). 

Regardless of the treatments, the chlorophilic and carotenoids levels were lower in the second year than the first one (**Figures 5B, C**). The RW irrigation increased chlorophyll and decreased carotenoid contents; the combination of both stresses (RW-RDI) strongly decreased the two pigments by in the first experimental year: 47.7% less chlorophyll and 27.0% of carotenoids than the rest of treatments. In 2018, a tendency to decrease both pigments by DW-RDI was observed. 

## Discussion

### Water Quality

Treated RW contain soluble minerals which depend quantitatively and qualitatively on the original source of the water and the type of treatment (Petousi et al., 2015). Our results were roughly in line with what we would expect from a properly performing secondary wastewater treatment plant. Although the EC and SAR values of RW were relatively higher than DW, they met the limits of the D.L. 185/2003. Besides, it is known that olive trees can tolerate irrigation water salinity of up to 5 dS·m^−1^ with a SAR of 18 (Tattini et al., 1992). The phytotoxic Cl^−^ and Na also were higher in RW, with respect to DW and the Cl^−^ levels exceed the limit. Moreover, RW contained quantities of nutrients as well as essential elements higher than DW source, although it also showed elevated levels of Al and Ni, according to Rosecrance et al. (2015).

### Effects of the Water Quality and Amount on the Olive Production Properties

In the first year of our experiment, trees were still under juvenile phase, giving a very low fruit yield response. In the second year, Arbosana trees started to bear, confirming what was well stated in the literature (Camposeo and Vivaldi, 2018). Water quality was key in terms of fruit yield. The higher yield obtained in RW full-irrigated trees was a consequence of the increasing in fruit weight and number due to the presence of nutrient elements. Thus, RW irrigation worked as fertigation. In the RDI treatments, the fruit yield was not affected by water stress although the percent of fruit set was higher and fruit weight was lower than in the control, mainly in DW-RDI, without any significant differences (**Figure 2**). Our results are in agreement with the finding of other researchers who reported that olive irrigation with treated wastewater significantly increased the fruit yield (Bedbabis et al., 2010; Bourazanis et al., 2016; Ayoub et al., 2016). A long-term experiment of 8 years conducted in Israel with “Barnea” and “Leccino” trees irrigated with saline wastewater of lower EC than ours (EC_w_∼1.7 dS·m^−1^) and fresh water reported that fruit yield was not significantly different among treatments in any individual season; however, the highest values of total yield in “Barnea” olives for the entire experiment was found in the wastewater treatment (Erel et al., 2019). . In other experiments carried out in Córdoba (Spain) under field conditions with Picual olive, two saline treatments (EC_w_ = 5 dS·m^−1^ and EC_w_ = 10 dS·m^−1^), without using wastewater from treatment plants, did not change annual yield in any of the 9-year studies (Melgar et al., 2009). In contrast, authors Gucci and Tattini, (1997) reported that a significant yield reduction occurs in olives cultivated under high-saline conditions (EC_w_ >7.5 dS·m^−1^). Ben-Gal et al. (2017) also informed that fruit yield decreased with increasing salinity in “Barnea” trees irrigated with water of different EC_w_ (from 2 to 11 dS·m^−1^). Other studies, such as Ben Ahmed et al. (2007) and Chartzoulakis (2011), reported that saline waters might reduce yield compared with good quality water. Concerning fruit characteristics like fruit weight, Bourazanis et al. (2016) found that treated wastewater produced heavier fruit than those irrigated with fresh water, as in our results. However, Melgar et al. (2009) showed that salinity did not affect fruit weight.

Regarding water amount, a decrease in biomass production due to deficit irrigation for many fruit trees does not necessarily lead to a parallel reduction in fruit yield because of changes in biomass partitioning between the different organs (Gucci et al., 2019). As a result, no reductions in yield have been reported for peach (Gelly et al., 2003), plum (Intrigliolo and Castel, 2010), almond (Stewart et al., 2011), pear-jujube (Cui et al., 2009), apricot (Perez-Pastor et al., 2014) when the stress applied during the irrigation season was moderate. In olive trees, the water volume can be reduced well below the level of full satisfaction of water needs with limited or no effects on fruit yield (Moriana et al., 2003; Gucci et al., 2007; Lavee et al., 2007; Gómez del Campo, 2013), as in our results.

As for ripening indices, the detachment index and the fruit firmness are usually used to detect the optimal harvesting time for cultivars with a long maturation period, as “Arbosana” (Camposeo et al., 2013). The lowest detachment index was found in RW-FI with values about 2.1 N·g^−1^, being this really positive for the mechanical harvesting efficiency, which is maximum (90–95%) when the detachment index is around 2 N·g^−1 ^(Farinelli et al., 2012; Camposeo et al., 2013). The RDI strategies did not affect the detachment index, unlike Rosecrance et al. (2015) who reported that the RDI had a lower fruit detachment force, contributing to greater percentage fruit removal. A low fruit firmness was presented by water stress with DW (DW-RDI), contrary to another study that did not observe differences on fruit firmness by deficit irrigation (Rinaldi et al., 2011). In the case of the RW-RDI, a low fruit firmness was not observed likely due to the salinity from RW. Regarding fruit WC, it is known that moisture levels below 50% and about 60% are difficult for the mill to extract the oil (Rosecrance et al., 2015). In our study, all treatments are within that range, although the RDI had the highest values, in contrast with Rosecrance et al. (2015) who found that water stress decreased fruit WC. Finally, crop load could explained the higher PI in 2017 (1.5; very low bearing year), with respect to 2018 (bearing year) since it is known that fruit ripening strongly depends on crop load, among other factors as the climatic conditions (Camposeo et al., 2013).

### Effects of the Water Quality and Amount on Oil Industrial Variables

In the process of oil extraction, the irrigation water amount was more decisive than water quality. The treatments under water stress (RDI) presented the lowest fatty yield and oil extractability, which is probably caused by the increase in humidity in the olive paste which hindered the oil extraction, as García et al. (2013) explained. Other researchers have also found that too high fruit humidity content decreases oil extraction (Gómez-del-Campo, 2013). The acidity levels, however, improved with the RDI.

Concerning oil yield, it is known that it is a function of the fruit yield as well as the percent of oil (Ben-Gal et al., 2017). In our study, the water quality was a more determining factor than the amount of water. The lowest values of oil content per dry or fresh weight were found in RW trees. Thus, although the RW-FI had the highest fruit yield, the oil yield did not improve, with respect to the control. So far, nothing has been published accordingly with our results about effects of RW-FI on the oil yield. Ayoub et al. (2016) did not find differences on oil content per dry fruit weight basis among fresh water and RW. Ben-Gal et al. (2017) cited that oil content in fruit is increased with the water EC during two years in Barnea trees irrigated with saline water. Bourazanis et al. (2016) also indicated that Koroneiki trees irrigated during two years with treated water produced more oil per tree than those irrigated with fresh water. Similar results have been also described by Segal et al. (2011), reporting a slight but non-significant increase in olive and oil yield in Barnea and Leccino. With regard both stresses combined (RW-RDI), there is nothing mentioned in the literature.

Moreover, as for water amounts, oil content dry weight-based slightly increased in the treatment with water stress (20% more in DW-RDI respect to the control), leading to the highest oil yield, despite its low oil extractability. Water stress could indirectly increase oil yield in many ways including: (1) improving the light environment for oil accumulation, (2) hastening fruit maturity at harvest, and (3) increasing fruit removal percentages from the trees (Rosecrance et al., 2015). In this work, only the first hypothesis would be possible. Trees growing under water stress (DW-RDI) had lower branches length than control trees (data not shown), and although light environments were not measured, these smaller trees likely had a greater proportion of the fruit exposed to high irradiance which likely contributed to increased oil yields. Other authors already found that to maximize olive oil yield, high irradiance is needed (Gómez-del-Campo et al., 2009; Cherbiy-Hoffmann et al., 2013).

There is dispersion in the literature about the effects of water stress on oil yield. Similar results to ours were found by a number of authors who reported curvilinear relationships between oil yields and water application, indicating that oil yields are maximized at water application rates below 100% of FI (Moriana et al., 2003). This is, the oil percentages can be increased with moderate water stress (Gucci et al., 2009; Caruso et al., 2013; Gómez-del-Campo, 2013; Rosecrance et al., 2015). Other authors cited the water volume can be reduced well below the level of full satisfaction of water needs with limited or no effects on fruit yield and oil yield (Gucci et al., 2007; Gispert et al., 2013; Gómez-del-Campo, 2013; Hernández et al., 2018).

Contrary to our results, in Fernández et al. (2013), a small reduction in oil yield by 26% was observed when the water applications were reduced by 72% in an olive orchard of “Arbequina.”

### Effects of the Water Quality and Amount on Oil Quality Parameters

In many parameters, differences between the two experimental years of the study were found as a consequence of the different duration of the RDI period as well as of environmental factors and fruit load, which are known to affect oil quality (Ayoub et al., 2016).

#### FA, Peroxides, K_232_, K_270_, Delta K Index

As for oil quality legal attributes, water quality (RW-FI and RW-RDI) did not affected the FA, according to Bedbabis and Ferrara (2018) who showed that acidity values were not significantly increased by saline water, as also reported by Bourazanis et al. (2016) using wastewater. However, the RW source accelerated the oxidation of the oil causing elevations in the peroxides and ΔK in 2017. Other works affirmed that these parameters were not affected by RW (Bourazanis et al., 2016).

The reduction of water amount combined with DW (DW-RDI), slightly decreased FA and peroxides, improving oil quality; however, it increased the K_232_ and K_270_ characteristics compared with the control, although only in the first year. A high value of these coefficients results in a lower resistance to oxidation of the oil and with a greater degree of oxidation. Changes in oil quality due to water deficit have been reported for many olive cultivars (Servili et al., 2007; Gómez-del-Campo and García, 2013; Caruso et al., 2014, Caruso et al., 2017; Hernández et al., 2018). Although most of these studies have shown that the irrigation regime had negligible or no effects on parameters as FA, peroxide values, spectrophotometric indices (K_232_, K_270_ and **Δ**K) (Gucci et al., 2019). 

In general, the values of FA, peroxides, and K_232_ and K_270 _for all oil samples examined here were lower than the maximum limits established by the cited EU legislation for the extra virgin olive oil (EVOO) category (FA ≤ 0.8; Peroxides Index ≤ 20; K_232 _≤ 2.5; K_270 _≤ 0.22; ΔK ≤ 0.01). 

#### Fatty Acid Profile

When we evaluated the FAME profile, oleic acid was the dominant acid in the olive oil obtained in all irrigation treatments (ranging from 69% to 71%), followed by palmitic (15.34–15.74%), linoleic (8.24–9.26%), and stearic acid (2.03–2.17%), with their concentration falling within the range of the values for characterizing it as EVOO.

The FAME composition was more affected by water quality than by water amount. Previous studies reported increases in palmitoleic (Ben Brahim et al., 2016) and linoleic (Bedbabis and Ferrara et al., 2018) acids in treatment irrigated with saline water, as in our results. The important increase (7–9%) of the linoleic acid was probably due to the fatty synthase enzymes stimulation as fruit maturity progressed in RW treatments. Since linolenic acid were not affected by RW, according to Bourazanis et al. (2016), the ω6/ω3 ratio of oil from RW treatments increased around 10%, as also Tietel et al. (2019) indicated. Furthermore, other important acids as oleic and stearic reduced their percentages when the olive trees were irrigated with RW.

Moreover, in our experiment the reduction of the water amount (DW-RDI) only affected a few minority fatty acids as C20:1, C22:0, and C24:0 and decreased the linoleic acid respect to the DW-FI, although only in 2017. Accordingly, Hernández et al. (2018) found a decrease in linoleic acid under severe RDI conditions. Moreover, several studies for many cultivars have shown that the water deficit had negligible or no effects on FAME composition (Gómez-del-Campo and García, 2013; Caruso et al., 2014, Caruso et al., 2017).

As for the effects of the combination of both stresses (RW-RDI) on fatty acid, we observed that the reduction of oleic acid and the increase of linoleic were more pronounced than in RW-FI. It is not possible to discuss these results with other work because, so far, nothing has been published about the combination of RW and RDI strategies.

The effects reported here regarding FAME profile did not show very great differences among treatment from an agronomical point of view. However, they have a large nutritional significance, since they reflect a trend of declining nutritional and health quality of olive oil as a result of irrigation with RW. Many of the health-promoting traits of olive oil are ascribed to its monounsaturated fatty acid content, mainly the oleic acid. This has been clinically proven to enhance cardiovascular health and improve blood-lipid profile, in addition to other metabolic-syndrome-related advantages (Tietel et al., 2019). In addition, the low levels of saturated fatty acids and the ω6/ω3 ratio play a key role in the bioactivity of olive oil as a functional food (Tietel et al., 2019). Specifically, higher ω6/ω3 ratios, as occurred in this experiment with the use of RW, increase the risk for obesity (Simopoulos, 2016).

#### Parameters of the Minority Unsaponificable Fraction  

Both water quality and amount affected the polyphenol levels, which play a role in the stress response and defense mechanism of the tree. In our experiment, saline RW (RW-FI) increased oil polyphenols , probably due to stress response to high salt levels, as reported by Chartzoulakis (2011). Salinity stress causes subsequent water deficit, which has been shown to be involved in the activation of phenylalanine ammonia lyase (PAL) (Ben Ahmed et al., 2009), a key enzyme directly involved in polyphenol biosynthesis in fruit, which causes an accumulation of phenolic compounds in the oil (Patumi et al., 2002).

Regarding RDI strategy (DW-RDI), it also improved the polyphenols content in oil, consistent with many studies (Gómez-del-Campo and García, 2013; Rosecrance et al., 2015; Caruso et al., 2017) due to water deficit enhanced synthesis of these compounds in the fruit, according to Alagna et al. (2012). Severe conditions trigger antioxidation mechanisms activated by the plant in response to oxidative stress, and hence accumulate in oil (Tietel et al., 2019). Others findings suggest that the catabolism of phenolic substances in the fruit is likely influenced by water stress too (Cirilli et al., 2017).Moreover, RW-RDI was the treatment that showed the greatest increases in polyphenols both years. As far as we know, it is the first time that data are reported respect to the combination of water and saline stresses and nothing has been published in the literature.

From the point of view of food quality, the irrigation effects on the polyphenols levels are relevant for oil sensory quality and for the health-promoting effects of the oil (Clodoveo et al., 2015; Tietel et al., 2019) as the prevention of the formation of cancer cells, so that European Food Safety Authority (EFSA) launched a specific health claim (EU, 2012). In addition, polyphenols are also key contributors to oxidative stability, mainly 3,4-DHPEA and its secoiridoid derivatives (Servili et al., 2004). Thus, irrigation with RW and RDI and mainly the combination of both might also greatly positively affect oil shelf life and nutraceutical claim.

Finally, the climatic pattern during ripening period could explain the strong increase of 25% of total oil polyphenols contents in 2018 with respect to 2017. Indeed the second year was colder than the former. In the literature, it has been stated that low temperature stimulates polyphenol accumulation in olive oil (Artajo et al., 2006).

Color is a basic attribute for determining the characteristics of olive oil although analysis of the pigmentation is not required in the corresponding EU Regulation. The trees full irrigated with RW increased chlorophyll levels in 2017, being a positive aspect since most consumers associate the presence of chlorophyll with quality. Nevertheless, our results are contradictory with other studies (Ghrab et al., 2014; Bedbabis et al., 2015). As for carotenoids, their levels decreased in RW-FI, as Ghrab et al. (2014) also cited.

In summary, the trees full irrigated with RW improved the fruit yield although it did not increase the oil yield since the oil content dry weight-based was lower than control trees. The changes in oil fatty acid composition of these trees demonstrated tendencies that are undesirable, including increased unsaturated acids, as well as the ratio ω6/ω3. The peroxides also increased. On the contrary, higher levels of the polyphenols in oil were presented. The deficit irrigation, with DW, did not affect the fruit yield, although there was an increase in the number of fruits which showed less weight and firmness during the RDI period. Despite the reduction of the fatty yield and oil extractability due to the high fruit WC, this treatment presented the highest oil yield since oil content fruit dry weight-based improved by 20%. Furthermore, there was a reduction in the acidity and peroxides and an increase in the polyphenols of the oil by water stress. Some negative aspect were also found: an increase in K_232 _and K_270_, although within the legal limit, and in some minority acid as C20:0, C22:0, and C24:0. Finally, the combination of RW and RDI neither reduced fruit yield. Besides, its fruits did not lose as much weight or firmness as in DW-RDI. However, although the fatty yield and oil extractability decreased less than in DW-RDI, the oil yield values of these trees under both stresses were the lowest compared with the rest of treatments since the low oil content fruit dry weight-based. As for the oil quality of RW-RDI, similar results as in RW-FI were observed, plus an important decrease of pigments in the first year. It is important to highlight also that the highest levels of polyphenols were displayed in this treatment. These aspects described about the combination of both stresses in this paper are reported in the literature for the first time. These findings could help optimize crop management of cv Arbosana in new olive cropping system, where environmental sustainability represent a key factor.

## Data Availability Statement

All datasets generated for this study are included in the manuscript/supplementary files.

## Author Contributions

Data curation: GV, CR-T, AP. Formal analysis: GV, AP, CR-T. Investigation: GV, CR-T, EN. Methodology: GV, FS. Project administration: SC, GV. Resources: SC, GV. Supervision: SC, GV, EN. Writing – original draft: CR-T. Writing – review and editing: SC, GV, EN.

## Funding

The research involved in this work has been supported by the EU and Water JPI for funding, in the frame of the collaborative international Consortium DESERT, financed under the ERA-NET WaterWorks 2014 Cofunded Call. This ERA-NET is an integral part of the 2015 Joint Activities developed by the Water Challenges for a Changing World Joint Programme Initiative (Water JPI), and “Fondo di Sviluppo e Coesione” 2007-2013 e APQ Ricerca Regione Puglia “Programma regionale a sostegno della specializzazione intelligente e della sostenibilita sociale ed ambientale e FutureInResearch.” 

## Conflict of Interest

The authors declare that the research was conducted in the absence of any commercial or financial relationships that could be construed as a potential conflict of interest.
